# Conflict in intensive care units: a conceptual framework for patient, family, team, and inter-service disagreement

**DOI:** 10.1186/s13054-026-06248-7

**Published:** 2026-08-15

**Authors:** Craig S. Jabaley, Zoe Brummell, Dominique D. Benoit, Nancy Kentish-Barnes, Victoria Metaxa

**Affiliations:** 1 Department of Anesthesiology, Emory University School of Medicine, 1364 Clifton Rd NE, Atlanta, 30322 GA USA; 2 Emory Critical Care Center, Atlanta, GA USA; 3 Department of Critical Care, University College London Hospitals NHS Foundation Trust, London, UK; 4 Department of Targeted Intervention, University College London, London, UK; 5 Department of Intensive Care Medicine, Ghent University Hospital, and Faculty of Medicine and Health Care Sciences, Ghent, Belgium; 6 Famiréa Research Group, Medical Intensive Care Unit, Assistance Publique–Hôpitaux de Paris, Saint Louis University Hospital, Paris, France; 7 INSERM UMR 1342, Institut de Recherche Saint-Louis, Paris, France; 8 Department of Critical Care, King’s College Hospital NHS Foundation Trust, London, UK; 9 Honorary Reader, King’s College London, London, UK

**Keywords:** Conflict, Intensive care unit, Interprofessional communication, Moral distress, Shared decision-making, Ethics consultation, Psychological safety

## Abstract

For clinicians, conflict in the intensive care unit (ICU) is both common and expected owing to the nature of critical illness and its treatment. Examination of conflict in this context has been conceptually diffuse. Studies of moral distress, psychological safety, ethical climate, and other fields offer valuable insights that can be challenging to synthesize cohesively, due to differences in concepts, methods, and disciplinary perspectives. This narrative review integrates this literature using a task-process-relationship framework from organizational psychology. Conflict in the ICU arises in two principal arenas: between clinical teams and patients, families, or surrogates, and within and across clinical teams. Patients who retain decision-making capacity are a distinct and often overlooked party to conflict, as the focus has primarily been on the families and surrogates who speak for those who cannot. The task-process-relationship framework suggests a recurring escalation pattern for both arenas. Genuine task disagreements about treatment, prognosis, or goals of care can become destructive in the presence of process gaps or failures. These include role ambiguity, inconsistent messaging, and perceived unfairness. From there, conflict can enter the relationship domain wherein mistrust or identity threat makes resolution far harder, and two implications follow. First, much of what clinicians may describe as a “difficult family” can often be a response to antecedent process failures rather than a purely interpersonal problem, emphasizing the importance of engineering robust processes and systems in the ICU to avoid conflict reaching the relationship domain. Second, family and team conflict are linked in that unresolved team disagreement can reach families as contradictory recommendations and mixed messaging. Challenging family situations can expose team misalignment and damage collegial relationships. Evidence-based interventions, organized by conflict type and escalation stage, suggest that the most promising investments are structural, namely those built into rounds, improving role clarity, ensuring pre-conference team alignment, and fostering ethical climate. When conflict becomes entrenched, formal supports such as mediation and ethics consultation are matched to the nature of the dispute. While disagreement in intensive care is inevitable, destructive conflict is not.

**Graphical abstract:**

**Figure:**
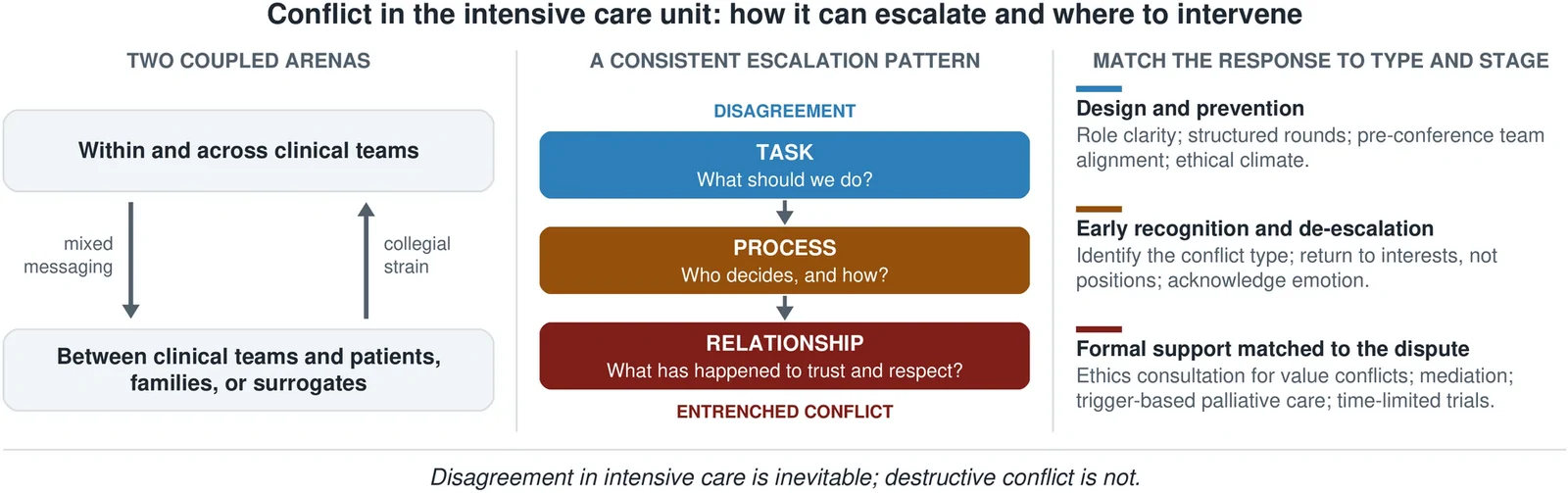

## Introduction

Conflict is a predictable part of work in the intensive care unit (ICU), which combines uncertainty, high stakes, distributed authority, and the pressures of limited time and resources. It is not only a problem of scarce resources; conflict persists in well-resourced units because its drivers are structural. In the CONFLICUS study of 323 ICUs across 24 countries, 71.6% of staff reported conflict in the previous week [1]. A scoping review of 15 adult ICU studies reached the same conclusion, with sources clustering around the care environment (staff shortages, limited resources, restricted visitation), end-of-life care, and management [2]. Despite this prevalence, the field lacks a single agreed definition of conflict, and studies vary widely in design and in how they detect it [3]. Conflict is often examined under adjacent labels rather than on its own terms, and the literature on conflict within and between teams is more scattered than the literature on conflict with families.

This review examines conflict in two arenas: between clinical teams and patients, families, or surrogates; and within and between clinical teams. Drawing on the organizational conflict literature, we define conflict as a perceived or actual incompatibility of goals, values, or interests that produces negative emotion or a sense of interference with one’s objectives [4–7]. This sets conflict apart from mere disagreement, which is a difference in judgment or preference that need not carry negative emotion or blocked goals. Conflict also arises between parties rather than within an individual, so it is inherently subjective [6]. Because the incompatibility need only be perceived, one party can feel it before it is expressed or recognized by others, and it need not be visible to outsiders [8].

Family conflict and team conflict are different arenas with similar dynamics, and we approach both through a structural lens rather than a pathology one. This structural lens does not assign fault: being a party to a conflict is a matter of position within a relationship, not necessarily culpability for its history [6–8]. Mistrust carried forward from prior healthcare experiences, for example, is an antecedent condition that lowers the threshold for conflict without itself constituting conflict with a clinician the family has just met; rather, a clinician becomes a party only when an incompatibility is perceived within the current relationship [8]. This framing does, however, assign prospective responsibility for preventing escalation and pursuing repair, which should be distributed in proportion to each party’s role, power, and capacity to act rather than owned by one “difficult” person. Prospective responsibility does not, however, guarantee resolution. Some conflicts remain unresolvable despite skilled effort, including facilitation and arbitration, and neither involvement in conflict nor its persistence constitutes personal or professional failure. In many healthcare cultures, disagreement is treated as something to avoid rather than a normal and sometimes useful part of complex decisions [9, 10]. Productive disagreement is defined as focused on the clinical task, occurring within a process the parties accept as fair, and leaving no lasting damage to the relationship.

## Methods

We conducted a narrative rather than systematic review because our aim was interpretive: to synthesize several distinct literatures (moral distress, psychological safety, ethical climate, negotiation science, and organizational conflict) into a shared framework rather than answer a single question suited to meta-analysis. Because the subject is cross-disciplinary, we searched PubMed/MEDLINE; PsycINFO and Business Source Complete (covering organizational psychology and management); HeinOnline (covering law); and Google Scholar through 31 March 2026, adding citation tracking and hand-searching of key reference lists [11, 12]. During peer review, supplementary targeted searches of the same sources were conducted through July 2026 to address reviewer comments. We favored primary studies and authoritative syntheses and checked the most load-bearing quantitative claims against their original sources [13]. Strength-of-evidence descriptors in the summary tables reflect our qualitative judgments of study design and directness rather than formal, protocolized ratings. As an interpretive synthesis, this review is intersubjective and cannot be reduced to the aggregation of comparable measurements [14]. We do not address conflict within families in depth, which is consequential but beyond the scope of this work.

## Framework for understanding ICU conflict

### Task-process-relationship framework of conflict

**Fig. 1:**
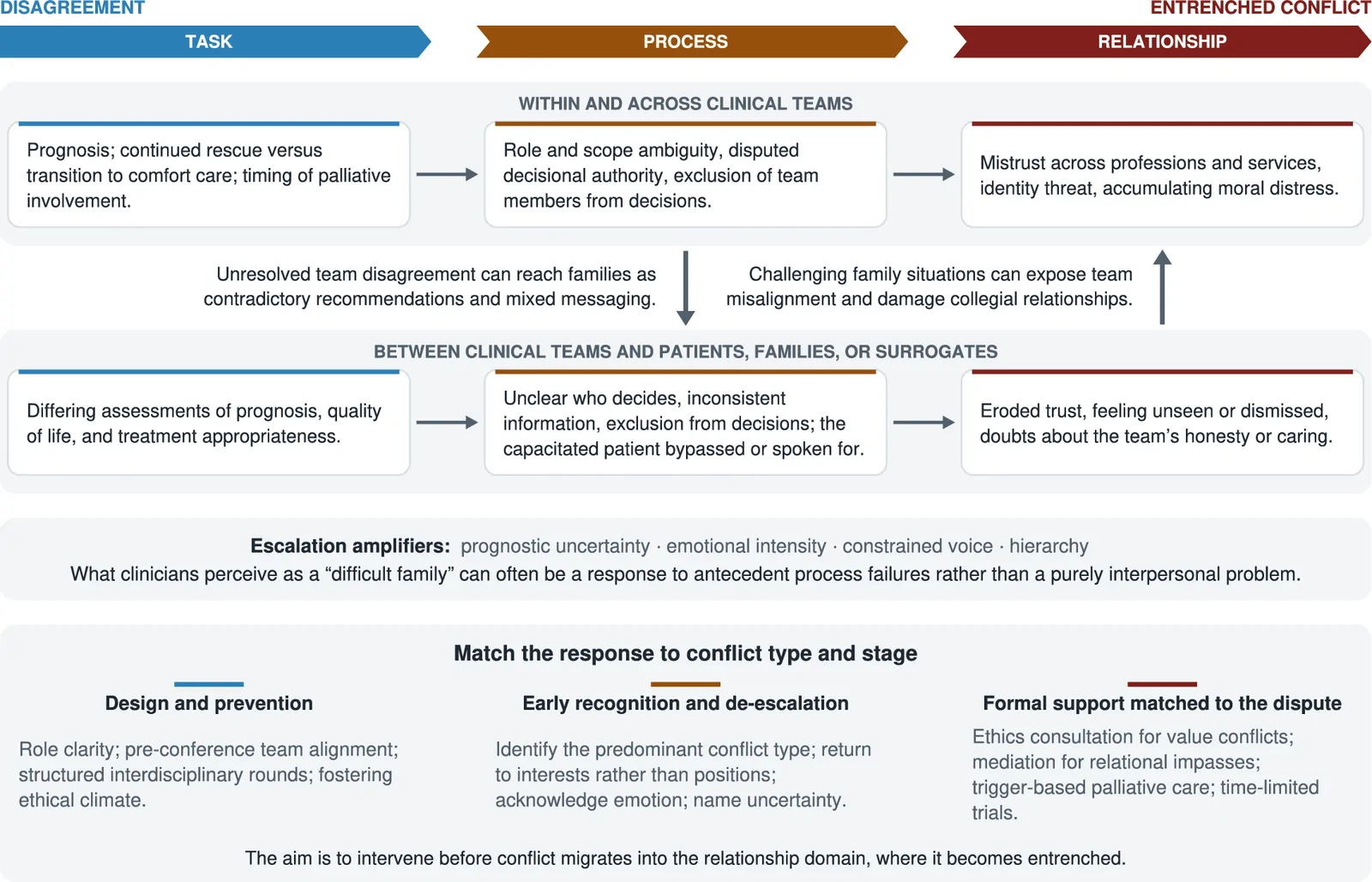
How conflict can escalate and spread in the intensive care unit, and where to intervene. Conflict can escalate from the task domain (what should be done) through the process domain (how decisions are made, and by whom) into the relationship domain (whether trust and respect remain intact) [4, 15, 16]. The same escalation operates in two arenas, which are coupled. First, unresolved team disagreement can reach families as contradictory recommendations and mixed messaging. Second, challenging family situations can expose team misalignment and damage collegial relationships. Cell entries are the authors’ synthesis, illustrated with representative examples from the literature reviewed. The framework is an organizing heuristic rather than a fixed sequence, because conflict types often combine, and process conflict can precede explicit task disagreement. For some patients and families, mistrust grounded in prior healthcare experience precedes the ICU encounter, and conflict can enter at the relationship domain rather than arriving there. Trigger-based palliative-care engagement is preventive when adopted as standard practice and is grouped with formal supports here for already-established disputes

The task-process-relationship framework offers a diagnostic map for how conflict escalates (Table 1, Fig. 1). It has not been validated specifically in intensive care, but it is well supported by research on work teams in organizational psychology and has been applied to healthcare team conflict in an integrative review [15]. We use it as an organizing heuristic whose value is to integrate otherwise siloed literatures and to generate testable predictions: that conflict tends to migrate from the task and process domains into the relationship domain; that process failures are a frequent and underrecognized driver of what presents as task or relational conflict; and that structural and interpersonal interventions act at different points on the same causal pathway.

**Table 1:** Three-type analytic framework for conflict

**Type**	**Core question**	**Intensive care examples**
Task conflict	What should we do?	Prognosis and trajectory assessment; treatment appropriateness and intensity; extracorporeal support candidacy; resuscitation preferences; antibiotic escalation or de-escalation
Process conflict	Who decides, and how?	Role authority and decision ownership; consultation and referral norms; family conference structure and scheduling; admission, discharge, and triage governance; surrogate identification and decisional burden^*^
Relationship conflict	What has happened to trust and respect?	Professional antagonism and interpersonal blame; family distrust of the clinical team; service rivalry and territorial behavior; identity threat and moral injury; “they don’t care about the patient”

^*^Where formal surrogate decision-making is not the default (e.g., best-interests or benefits-based models in the United Kingdom and elsewhere), the analogous process question is how families and clinicians are jointly involved in those determinations

Primary analytic framework adapted from Jehn (1995) and de Wit, Greer, & Jehn (2012) [4, 16]. Many intensive care unit conflicts begin in the task or process domain and become destructive when they migrate to the relationship domain

The framework distinguishes three kinds of conflict. *Task conflict* concerns the substance of a clinical decision: What is the prognosis? Is this treatment appropriate, and can it achieve its goal? *Process conflict* concerns how decisions are made and who makes them. When a patient cannot participate, who decides, and through what process? How are differing views within a team reconciled? The answers vary across jurisdictions and carry moral, ethical, and legal dimensions. *Relationship conflict* involves damage to trust, respect, or collegial regard, such as a sense that others do not care about the patient, that a colleague is incompetent, that clinicians are dishonest, or that another party is difficult.

These types often combine, and their harms intensify when they do [4, 5, 16]. Relationship conflict is the hardest to resolve, because the parties then read each new interaction through the lens of mistrust. Its emotional register is broader than mistrust alone, extending to anger, fear, and grief, each carrying its own moral weight. Anger has been described as a moral emotion that signals a perceived wrong, though in a relationship it is seldom cleanly separable from mistrust and may persist as a self-protective response to prior harm [17, 18]. Theories of relational identity hold that threats to a person’s professional role or moral integrity can act as high-intensity relationship conflict, a pattern seen in ICU studies [19–22]. Conflicts experienced as identity threats behave differently from ordinary task disagreement: they escalate faster, leave moral residue that builds across episodes, and contribute to burnout and intention to leave [23]. Mitigation must therefore acknowledge the threatened standing before returning to the substantive question, because the dispute has shifted from what should be done to whether the dissenting party is respected.

Several factors amplify escalation: prognostic uncertainty, emotional intensity, constrained voice, and hierarchy. Others are antecedent conditions that increase vulnerability without themselves constituting conflict, including prior negative healthcare experiences, limited cultural or linguistic concordance, and pre-existing distress [8]. Mistrust of this kind is measurable among ICU surrogates and is carried into new relationships: negative healthcare experiences, whether the patient’s own or a family member’s, are associated with lower trust in physicians who had no part in them [24–26]. The two layers are also separable in time. In a pilot ICU cohort, surrogates’ health-system distrust declined over the stay while trust in the treating physician moved independently [24]. In a multicenter ICU cohort, surrogates’ trust in the treating physician increased in association with shared decision-making behaviors, highlighting the importance of perceived conduct [27]. Failing to speak up is common and contributes to communication failures [9, 10]. Hierarchy also shapes who perceives conflict in the first instance. Physicians consistently report better collaboration than nurses do, both in the ICU and more broadly, across Europe and the United States (US) [28–31]. Physicians therefore likely underestimate the conflict that others experience, and conflict that goes unrecognized cannot be addressed. Detecting it reliably requires structured input from every team member.

Equity impacts all three domains rather than functioning as a separate conflict category. Social hierarchies shape whose account is treated as credible, who receives comprehensible information, who participates in decisions, and whose distress is read as threatening; however, the locally salient axes vary by setting and have been described as including race, ethnicity, Indigeneity, caste, class, religion, migration status, and language [32–37]. Healthcare is also experienced differently along dimensions beyond these, including physical and intellectual disability, sexual orientation, and housing status [38–41]. These characteristics do not themselves cause conflict; rather, inequity arises when institutions and interactions convert them into differences in voice, trust, information, or exposure to coercive responses. Much of the mechanism-level evidence comes from US studies of Black patients, which we draw on subsequently [42].

### Complementary perspectives

Several further lenses complement this framework. Negotiation science distinguishes interests, the underlying needs and values a party holds, from positions, the specific demands they state [43]. A demand that “everything be done,” for example, is a position that may rest on an interest in knowing the patient will not be abandoned; interests can be compatible even when positions collide. Principled negotiation has been criticized for underplaying real or perceived power asymmetries, which matter in clinical settings where clinicians hold authority from both their role and their expertise [44, 45]. Procedural-justice research shows that people are more satisfied with decisions when the process is fair, transparent, and respectful, even when they disagree with the outcome [46]. Work on psychological safety and relational coordination identifies the conditions that let teams raise and work through disagreement: shared goals and knowledge, mutual respect, and frequent problem-solving communication [47, 48].

A related lens, less empirically grounded but practically useful, separates basic human needs and core values, such as the competing interests at stake in intensive care and respect for the patient as an individual, from a more superficial layer of norms and expectations [49, 50]. What is labelled a “values conflict” is often a clash at this superficial layer, and resolving it usually means returning the parties to the deeper one. This distinction can also blur ethical principles. Clinicians may continue treatment they judge non-beneficial, with family insistence the most cited reason, and clinicians’ own reluctance can also contribute [51, 52]. Interpreted through this lens, beneficence is recast as not disappointing the family, which privileges the surface relationship over the deeper obligation. Families, in turn, may treat autonomy as entitling them to any treatment they request [53]. The underlying principles are more precise: autonomy is the right to make informed, voluntary choices about one’s own care (including refusal), not a right to oblige clinicians to provide treatment that is harmful or cannot achieve its goal. Beneficence and non-maleficence remain distinct obligations, not displaced by respect for autonomy [54].

### The value of structured task disagreement

Task disagreement includes differences over diagnosis, prognosis, or treatment, and in principle it can improve a difficult decision because it brings different knowledge and experience to bear. However, the empirical support is weaker than often assumed. Meta-analyses find only a weak overall association between task disagreement and team performance, and the benefits concentrate under specific conditions: when task disagreement is weakly coupled to relationship conflict, when the team sits higher in the organizational hierarchy, and when the outcome measured is the quality of the specific decision rather than the team’s broader efficiency or output [4, 16]. The productive potential of task disagreement therefore has to be created deliberately, not assumed. Structured dissent guards against premature consensus, a known risk in high-stakes team decisions [55, 56]. The closest clinical evidence comes from diagnostic reasoning: prompting physicians to weigh alternatives rather than settle on the first plausible answer improved accuracy on bias-prone cases [57].

At the team level, the strongest outcome signal comes from team-training bundles: an ICU crew-resource-management cohort reported fewer serious complications and lower mortality, and a large medical team-training program was associated with lower surgical mortality [58, 59]. Because these are bundled interventions, the contribution of structured disagreement specifically cannot be isolated, and ICU results are mixed overall, including a multisite controlled trial that found no effect on clinician behavior or patient outcomes [60, 61]. How disagreement is handled matters as much as whether it is voiced. The practical lesson is to structure disagreement, not merely tolerate it: rounds that solicit dissenting views, a designated devil’s-advocate role, and a team culture that separates challenging an idea from challenging a person. Formal devil’s-advocacy does improve decision quality in controlled studies, but that evidence is largely non-clinical, so its clinical value is best seen as mechanistically motivated rather than demonstrated [62].

## Conflict with patients, families, and surrogates

Families and surrogates are present at nearly every ICU bedside. They bring knowledge of the patient’s values, preferences, and subtle changes that deepen the team’s understanding. Engaged compassionately, their involvement strengthens shared decision-making. Conflict arises when task-level misunderstandings or process-level gaps go unaddressed, and early attention to communication and trust can keep it from escalating.

Conflict in this arena is heterogeneous, and a two-axis taxonomy—who is in conflict, and what about—helps to organize its distribution (Table 2). Its drivers have a previously suggested “differential diagnosis,” with contributors spanning the family, the clinician, and the institution rather than residing in the family alone [63]. Team-family conflict is usually the largest category and divides about evenly between poor communication and disagreement over life-sustaining treatment, so roughly half presents as a process problem rather than a clash of values [1, 64–67]. Prevalence also depends on who is asked: physicians report conflict far less often than surrogates or nurses, so single-informant figures are understated [68].

**Table 2:** A two-axis taxonomy of conflict around the critically ill patient

Party^*†^	**Task**	**Process**	**Relationship**
Team-family: 57% [64]; 17% [1].^‡^	LST-preference clash 44% [64]; physician-surrogate prognostic discordance 53% [67]	Poor communication 44% (difficulty comprehending prognosis 19%, language barriers 13%) [64]; decision-maker unavailable/unable 15–16% [64]; ~ half of families comprehend poorly [66]	Coping or emotional reactions 15%; family mistrust of clinicians 3%; religious-cultural 6% [64]
Intra-team: 31% [64]; nurse-physician the most common pair (33%) [1]	Care or management disagreement 55% (pain/sedation 9%, prognosis 5%); LST 7% [64]	Poor communication 16%; lack of leadership 9%; lack of coordination 7%; nurses excluded from decisions 9% [64]	Diffuse; inter-physician friction and burnout [20]
Team-patient: 17% [1]	Capacitated patient rejects a recommendation: under-studied	Patient bypassed or spoken for; refusals reframed as capacity questions [139]	Patient feels unheard: evidence gap
Intra-family: 12% [64]	LST 57%; other care-plan decisions 37% [64]	Communication problems 13%; surrogate or proxy appointment 10% [64]	Internal animosities 3% [64]

^*^ Overall prevalence: 71.6% of ICU staff reported ≥ 1 perceived conflict in the preceding week (323 ICUs, 24 countries) [1]

^†^ Conflict is systematically under-detected by any single informant: physicians identified conflict in 27.8% of cases versus 42.3% by surrogates, and nurses identified it more often than physicians [64, 68]

^‡^ A passive surrogate decision-making role is associated with anxiety (88%) and depression (50%) [298]; surrogate decisional conflict is common and is reduced by prior advance care planning [299]

Conflict in the intensive care unit (ICU) is classified along two axes: the parties involved (rows) and the predominant source of the disagreement (columns; task, process, and relationship domains as defined in Table 1). In this synthesis, cell entries give the proportion of conflicts of each party-type attributable to each source and are drawn from the cited primary studies. Source categories reflect the original studies’ labels; emotional reactions and mistrust may reflect pre-existing vulnerability to conflict rather than conflict itself. Note that percentages derive from two cohorts with different denominators and are not directly comparable. The figures attributed to Studdert et al. [64] partition that study’s conflicts into mutually exclusive party categories summing to 100% (team-family 57%, intra-team 31%, intra-family 12%, totaling 100%). The figures attributed to the CONFLICUS study [1] are the proportions of conflict-reporting respondents who experienced a conflict with each party, and because a respondent could report conflict with more than one party, these overlap and do not sum to 100%. LST, life-sustaining treatment

### The institutional position of families

Surrogacy is an institutionally constructed role. Over recent decades, families have been redefined from visitors to active partners and, when the patient cannot communicate, to surrogate decision-makers, with substantial international variation in their scope and authority [69]. The position is structurally asymmetric: families take responsibility for consequential decisions without the clinicians’ knowledge base, professional framing, or, in some systems, formal authority. On knowledge, a prospective study found that 19% of surrogates poorly comprehended ICU care and nearly half had poor knowledge of the patient’s care, and comprehension itself shaped willingness to share in decisions [70]. On framing, surrogates often meet the situation through technical language, such as probabilities, organ-system trajectories, and judgments of “appropriateness,” even when clinicians work diligently to translate these concepts. These asymmetries can generate disagreement and distress and are better viewed as structural features of how decision-making is organized than as individual misunderstanding, which helps explain why intensive communication or facilitation interventions have not reliably improved family psychological outcomes [71, 72].

The family’s institutional position also varies by jurisdiction. For example, in the US, Canada, and Australia, statutory or common-law surrogate hierarchies usually designate family members as default decision-makers when a patient lacks capacity, even without a prior legal instrument. The United Kingdom and many other jurisdictions use best-interests, benefits-based, or similar models, in which clinicians make the decision on the patient’s behalf after consulting those close to the patient, unless the patient appointed a Lasting Power of Attorney, made a valid advance decision, or has a court-appointed deputy [73]. Continental European, Israeli, and Asian systems fall at various points along this spectrum, and the ETHICUS-2 cohort documented wide cross-national variation in nurse involvement in end-of-life decisions [74, 75]. Kinship structures can also diverge from the legal construct of next of kin: in Australian Indigenous communities, the appropriate spokesperson may be a community-nominated Elder or a specific relative outside the nuclear family, so a decision process built around legal next of kin can itself misidentify who should speak [76]. India illustrates the same gap from another direction: with no authoritative legal definition of next of kin for healthcare decision-making, individual hospitals improvise their own surrogate hierarchies [77].

We use “surrogate” as shorthand, but these asymmetries apply broadly. Even where families are not formal decision-makers, they are routinely drawn into consequential decisions, asked to interpret the patient’s prior wishes, and exposed to the same gaps in knowledge, authority, and framing. The legal model changes who has formal responsibility, not the family’s role in experiencing or contributing to conflict. The framework’s application here should be read as jurisdiction-agnostic, while specific examples reflect the context in which they were generated.

### Applying the framework to conflict with patients and families

For families and surrogates, task conflict centers on what should be done: differing assessments of prognosis, quality of life, and treatment appropriateness. Such disagreements often look value-based, but prognostic misunderstanding runs both ways. Surrogates can misread clinician estimates, and clinicians can misread what families believe and prioritize, so both may act on false premises about the other [78].

Cultural and religious commitments shape these interactions in ways a biomedical frame sometimes accommodates poorly. Religious belief can be associated with preferences for intensive treatment near death, but the association is heterogeneous; some surrogates who believe in miracles still choose comfort-focused care [79, 80]. An apparently fixed demand may rest on a religious or cultural commitment worth eliciting: in a multicenter analysis of 249 recorded goals-of-care conversations, surrogates raised religious or spiritual concerns in 16% of meetings, and clinicians usually redirected to clinical topics or acknowledged the concern without exploring it [81]. When spiritual frameworks go unaddressed, families can feel unseen or dismissed [82]. Mitigation calls for explicit process scaffolds: structured spiritual care, involvement of chaplains or community leaders, and clarifying decision-making roles.

These cultural and religious patterns are not only individual; end-of-life practice itself varies structurally across regions. Across European ICUs, the share of dying patients in whom life-sustaining treatment is actively withdrawn follows a stable north-to-south gradient with 47% in the north, 34% in central Europe, 18% in the south, which was documented two decades ago and since reaffirmed [74, 83]. Comparable variation is documented well beyond Europe, in East Asian, South Asian, Middle Eastern, and sub-Saharan African ICUs, and it tracks law, religion, and family authority more than clinical need; active withdrawal of life-sustaining treatment accounted for only 7% of end-of-life decisions in an Indian ICU, against roughly 45–80% in Western series [84–90]. Similar regional divergence is evident in Latin American end-of-life care beyond the ICU [91]. Attitudes toward patient autonomy track region and culture more than individual religiosity [92]. Consequently, when cultural differences surface as team-family conflict in multi-ethnic settings, it is co-produced with the structural features of intensive care and cannot be located in “the family’s culture” alone [33].

Process conflict often runs ahead of explicit task disagreement. Even where formal surrogate decision-making is not the norm, families are drawn into decisions for incapacitated patients and may be unsure who decides, receive information inconsistently, and feel acute process-related anxiety. The burden is real: most surrogates report negative psychological effects from the role, and one in five have moderate-to-strong regret at six months [93–95]. It worsens when the team has not resolved its own disagreement before presenting options. Bruce et al. described a “pas-de-deux” in which unresolved team disagreement can shape goals-of-care conversations, straining both the decision and clinicians’ sense of integrity [21].

A growing body of qualitative work reframes much of what clinicians call “family conflict” as a reasonable response to institutional failures across all three domains (Table 3). The label “difficult” deserves the same scrutiny. It has long been understood to describe an encounter, not a person, and usually signals a strained interaction [96]. Empirically, perceived difficulty tracks clinician and system factors and is reported more by clinicians who are burned out or dissatisfied [97, 98]. Because the label reflects perceived ill-treatment as much as any family trait, the obligation is to address that perception rather than manage the “difficult” party [99]. Stigmatizing descriptors entered in the medical record can propagate to other clinicians, negatively impacting their attitudes and care, and portraying the patient or family as difficult is a documented category of such language [100, 101]. In US cohorts, such labels are applied unequally, with Black patients carrying roughly 2.5 times the odds of negative descriptors, the drivers of which are under-studied [102, 103]. Labeling a patient or family as “difficult” risks conflating transient interactional difficulty with a durable attribute; instead, describing the specific behavior and its context is the better practice. Unsuccessful communication is a common denominator in such disagreements [104]. Family-level complexity (e.g., severe grief, family-system dysfunction, psychiatric comorbidity, genuinely irreconcilable values) does sometimes contribute [53, 64, 105, 106]. A dedicated taxonomy of end-of-life family conflict maps these intra-family drivers in greater detail, identifying prior family conflict, communication constraints, and assertions of control as the strongest predictors [107, 108].

**Table 3:** The clinician-family perceptual gap: what clinicians perceive versus what families experience

Domain	Study	Clinician perception or behavior	Family/surrogate experience	Framework interpretation
Prognostic understanding	Vick et al. [78]	Families misunderstand prognosis and are not ready to accept the clinical reality	Clinicians also misperceive surrogates’ prognostic estimates	Apparent value conflict may partly reflect mutual task-level misunderstanding; not all discordance is miscomprehension
Spiritual and cultural values	Ernecoff et al. [81]; Akeke et al. [82]	Clinicians redirect to medical topics when families raise religion; less engagement with Black families’ spiritual concerns	Surrogates raise religious considerations in 16% of goals-of-care meetings; religion is central to coping across racial groups	Clinician disengagement can convert resolvable task conflict (values exploration) into relationship injury (feeling dismissed)
Communication adequacy	Anderson et al. [109]; Moss et al. [110]	“Family misunderstanding” or difficulty accepting prognosis	Surrogates identify inconsistent communication across clinicians and teams as a concern, among others	Process failure (fragmented messaging) can generate what appears to be task conflict (goals disagreement)
Surrogate moral burden	Schenker et al. [112]	Family is “refusing to let go” or “in denial”	Surrogates describe painful intrapersonal moral struggle between honoring patient values and not wanting to feel responsible for death	Clinicians may misinterpret intrapersonal surrogate distress as task-level obstinacy
Conflict prevalence	Schuster et al. [68]	Physicians perceive conflict in 27.8% of interactions	Surrogates perceive conflict in 42.3% of the same interactions	Process opacity and emotional neglect create latent conflict
Racial equity	Ashana et al. [113]; Ray et al. [116]	Clinicians may perceive “difficult” or “demanding” families	Black caregivers receive less emotional support and prognostic information; describe burden of reframing communication	Systematized process unfairness and relational failure attributed to family characteristics
Language and access	Thornton et al. [114]; Espinoza Suarez et al. [115]	Standard conference conducted with interpreter	Interpreter-mediated conferences include 45% less clinician speech and support; linked to impaired autonomy and unmet end-of-life wishes	Structural communication deficit creates process inequity
Pandemic context	Kentish-Barnes et al. [300]	Visitor restrictions as infection control necessity	Families describe difficulty building clinician relationships, powerlessness from physical separation	Institutional process (visitor policy) can generate relational damage

Even so, process failures are a common and actionable driver. In qualitative studies, families and surrogates themselves report intrapersonal value tensions alongside process breakdowns, such as inconsistent information, feeling excluded from treatment decisions, and communication that erodes over time, as central to their distress [109–112]. What clinicians may interpret as “refusing to let go,” families experience as a moral struggle between honoring the patient’s values and not feeling responsible for the death [112]. For families from marginalized groups, gaps in institutional trustworthiness, racially patterned communication, and language barriers can turn structural inequities into relationship conflict [113–116]. In the best-studied setting, the US, Black caregivers receive less emotional support and less medical information in family conferences, and interpreter-mediated conferences contain less clinician speech and fewer supportive statements [113, 114].

The same mechanisms recur across health systems. Among culturally and linguistically diverse families in ICU end-of-life care, communication challenges, with professional interpreter use as low as 5–6% despite availability, combine with misaligned expectations and unilateral clinician decision-making as recurrent sources of conflict [32]. In a cohort spanning all 23 Victorian public ICUs, formal identification of interpreter need was associated with lower adjusted mortality, whereas a non-English language preference without that flag carried higher mortality, suggesting that recognizing language need, rather than language difference itself, tracks with outcomes [117]. In a cohort spanning seven ICUs in the US, patients with limited English proficiency transitioned to comfort-focused care more slowly and were more often physically restrained [118]. Norwegian immigrant families, for their part, describe filtering what the patient is told, inverting the usual frame in which clinicians control disclosure [119]. In New Zealand, nurses describe Māori end-of-life care as hinging on the inclusion of whānau, the extended family and kinship network central to Māori life and often broader than the Western “nuclear family,” which can be obstructed by default ICU processes built around small gatherings of legal next of kin [120]. In Indian ICUs, where clinicians overwhelmingly identify financial constraint as the leading driver of discharge against medical advice, treatment limitation occurs mostly through such unplanned terminal discharges, which leave the patient without palliative care and the family without support [35, 77]. Families generally perceive more conflict than physicians do in the same encounters, so process opacity can create the experience of conflict even when overt disagreement is limited [68].

These patterns reframe how trust should be understood. Trust is often presumed the default and mistrust a deviation to be corrected, but for many patients and families mistrust is an expected, historically grounded response rather than an error [121]. Accordingly, group-based medical mistrust is measurable and predictive [122, 123]. Experiences of racism in serious illness, particularly being disbelieved, deepen mistrust and degrade communication [124]. The same dynamic has been reported during Indigenous critical care in Australia, where clinicians describe a pre-existing, population-level trust deficit, shaped by colonization and prior negative healthcare experiences, and name establishing trust as foundational to care [76]. Indigenous patients themselves describe racism and culturally unsafe care as grounds for mistrusting and avoiding health services [125]. The constructive move is to shift the target from the family’s trust to the clinician’s and institution’s trustworthiness, which must be both sought and deserved [126]. Two cautions are worth noting. First, mistrust is real but not conclusively established as the proximate cause of all disparities, since adjusting for it did not narrow gaps in advance-care-planning completion [127, 128]. Second, treating mistrust as a fixed attribute risks disengagement; however, clinicians should remember that trust remains buildable within the current relationship, as described above [27].

### The patient who retains capacity

This review emphasizes patients who lack capacity, because many critically ill patients cannot take part in their own decisions [129–131]. That emphasis can obscure conflict involving patients who keep partial or fluctuating capacity. Although formal assessment identifies far fewer capacitated patients compared to physician judgment alone, policy statements suggest that at least a quarter of ICU patients remain able to take part in aspects of their care, and the move toward lighter, guideline-recommended sedation is enlarging that group [132–136]. When conscious patients can communicate, conflict shifts toward being bypassed or spoken for. Clinicians can infer patient preferences poorly even outside ICU: in two often-cited studies, physicians were unaware of their patients’ wish to avoid resuscitation in about half of cases and could not reliably predict the same patients’ choices after discussing them [137, 138]. When a conscious patient’s wishes diverge from a surrogate’s or the team’s, the disagreement is at risk of being reframed as a question about capacity rather than considered on its merits [139]. That such an increasingly common conflict remains scarcely studied is perhaps itself a finding: lighter sedation is expanding the conscious ICU population faster than the field has built the tools, data, or norms to honor those patients’ voices [140–145].

### Drivers and management

One under-studied structural driver of family expectations is inter-hospital transfer. Transferred patients are, as a group, sicker and fare worse than those admitted directly [146, 147]. Yet, families often read transfer itself as a signal that survival is achievable, and surrogates rate a better chance of survival as a reason for transfer more strongly than the physicians arranging it [148]. Goals-of-care discussions are documented before transfer infrequently and usually happen afterward, often close to death, and transfer is seldom treated as a preference-sensitive decision [147, 149, 150]. Transfer therefore plausibly seeds an expectation-reality gap that later emerges as conflict, though this chain is inferential and remains a research priority [151].

Managing family conflict starts with identifying whether the impasse is mainly task, process, or relationship. If task conflict predominates, clarify the specific decision, make uncertainty explicit, separate prognostic from values questions, and consider time-limited trials. If process conflict predominates, align the team before meeting the family, state decisional authority transparently, and structure conferences with documented follow-up, drawing on integrated frameworks for family communication across the ICU stay [152]. When the relationship has ruptured, acknowledging emotion and arranging formal facilitation (ethics consultation, mediation, palliative care, or chaplaincy) become essential, matched to the nature of the dispute [53, 153, 154]. Even when family conflict seems primary, team disagreement is often present beneath it, and a complex family situation can in turn strain collegial relationships.

## Conflict within the ICU and across other teams

The same task-process-relationship dynamics that drive family conflict operate within and across clinical teams, but the literature is fragmented across adjacent constructs and under-recognized (Table 4). On the whole, evidence-based strategies to reduce team conflict, including at the end of life, are lacking [155]. Team conflicts arise both within and across professional boundaries, with consequences for burnout, turnover, and the consistency of what families are told.

**Table 4:** Conflict-adjacent literatures capturing conflict within and between clinical teams

Literature	What it measures	What it reveals about conflict	What it misses
Moral distress	Emotional burden of constrained moral agency when clinicians believe the right course of action is blocked [23]	Unresolved disagreement about treatment appropriateness; accumulation of moral residue across episodes (crescendo effect)	The disagreement itself and the other party’s perspective; distinction between task, process, and relationship dimensions
Psychological safety	Perceived climate for interpersonal risk-taking; willingness to voice concerns without fear of reprisal [47]	Whether dissent is voiced or suppressed; hierarchy’s role in silencing lower-status team members	Content and resolution of specific disagreements; whether voiced concerns lead to change or are ignored
Ethical climate	Shared perceptions of ethical decision-making quality: mutual respect, open reflection, trust [31, 228, 234]	Quality of interdisciplinary deliberation; whether the unit processes moral disagreement constructively	Specific conflict episodes and their trajectories; how climate shapes individual conflicts
Collaboration	Teamwork quality; interprofessional communication and coordination ratings [28–30]	Communication gaps and decision-process problems; the perceptual gap (physicians rate collaboration far higher than nurses)	When high “collaboration” scores mask conflict avoidance rather than genuine resolution
Perceived inappropriate care	Clinician judgment that care does not serve the patient’s interests or is disproportionate to expected benefit [160]	Task disagreement about treatment goals and intensity; moral burden on dissenting clinicians	Process and relational dimensions; whether disagreement was voiced or suppressed; the family’s perspective
Speaking up / silence (voice)	Willingness to voice safety concerns and professional dissent; barriers to speaking up [9, 10]	Hierarchy-driven suppression of disagreement; speaking-up climate predicts voice behavior; healthcare workers frequently withhold safety-relevant concerns	What happens after concerns are voiced; whether speaking up resolves or escalates the underlying conflict

Much of the intensive care team-conflict literature is dispersed across these adjacent constructs, none of which use “conflict” as their primary index term. Such fragmentation creates the false impression that intrateam conflict is understudied. This author-developed synthesis highlights how these literatures, when viewed as part of conflict research, reveal a substantial and conceptually coherent evidence base

### Conflict across interprofessional boundaries

The nurse-physician boundary is the most documented form of team conflict and the single most common ICU conflict type, as demonstrated in the 24-country CONFLICUS study [1]. ETHICUS-2, across 36 countries, found nurse involvement in end-of-life decisions varying between regions, with physician-nurse agreement ranging from about 40% to 100% [75]. The gap has two easily conflated layers. The first is structural: bedside nurses often cannot join family conferences because of workload, acuity, short staffing, or meetings scheduled without coverage or during handoff. In one pediatric ICU, 98% of nurses thought they should attend family conferences but only 17% of those invited could do so consistently, and in a multicenter adult sample 45% rarely or never participated [156, 157]. The second is cultural: nurses are not always asked for input and may be unsure of their role when present. The balance shifts by setting; in well-resourced academic units, physicians not asking (60%) and limited training (66%) outweighed time pressure (10–19%) [156, 158]. Either way, weaker nurse-physician collaboration tracks with higher nurse moral distress and with perceived inappropriate care, which is inversely related to nurse involvement in end-of-life decisions [159, 160]. Such divergence is not limited to Western settings: in a Japanese survey, ICU nurses required a higher predicted survival than surgeons or intensivists before supporting emergency surgery for a patient with ambiguous wishes [161]. Consistent with this, conflict is less frequent where symptom control is shared and units hold routine staff meetings, which are structural fixes that embed the nurse rather than relying on individual voice [1]. Cross-cultural conflict can also manifest within teams, not only in their interactions with families. Norwegian ICU nurses described their professional norms (e.g., total responsibility for the patient’s care, comprehensible information, an orderly clinical environment) as colliding with families’ culturally grounded expectations of participating in care, and they struggled to judge unfamiliar expressions of pain when assessing the need for analgesia [162].

The bedside team is also changing in ways that create new boundaries. Nurse practitioners and physician assistants are now in roughly 72% of US adult ICUs, more common than physicians-in-training (about 52%), and these advanced practice providers (APPs) are established, effective team members [163, 164]. Their growth, driven partly by trainee duty-hour limits, has outpaced evidence on how mixed physician-APP-trainee teams negotiate roles, and unclear boundaries are a recognized source of conflict. The signal is clearest among trainees: 31% of surgical residents said APP presence detracted from their ICU experience, strongly associated with feeling that nurses routed clinical issues preferentially to APPs, and two-thirds of emergency medicine residents reported a negative effect on their education [165, 166]. Underlying this is ambiguity about who is responsible for what; ICU stakeholders may hold unclear or divergent expectations of trainees, and the field is still defining how far APPs substitute for, collaborate with, or teach physicians-in-training [167, 168]. This is an emerging process conflict over role and scope, largely absent from the foundational ICU conflict literature predating the workforce shift [1].

### Conflict with surgeons and other physicians

Surgeon-intensivist relationships can be conflict-prone. Ethnographic and survey research describes a strong surgical orientation toward postoperative rescue, marked by a personal commitment to see the patient through [169–171]. In the US, this has been termed “surgical buy-in,” wherein proceeding to surgery is treated as an implicit agreement to accept postoperative life support [172, 173]. In a national survey, 62% of surgeons endorsed creating such an informal contract, and many were reluctant to withdraw life support afterward, especially after a complication [173, 174]. The emotional burden of a complex postoperative course can be heavy; among surgeons it most often involves guilt and anxiety, with a risk of clinically or emotionally avoidant behavior [175–178].

The impulse to rescue, and the emotions that follow when rescue is impossible, are well described among clinicians who care for the seriously ill, reflecting both genuine commitment and the fear of failing the patient [51, 52, 179, 180]. Disagreement arguably reflects role tension more than individual temperament, especially given that the “intensivist” role is entered by several routes, including surgical training [181–185]. The operating surgeon bound by a covenant to a particular patient is conceptually distinct from the surgically trained physician practicing as an intensivist, a distinction the conflict literature rarely makes [186]. Surgeon-intensivist disputes can reflect two defensible claims: the surgeon’s claim to postoperative authority, grounded in a fiduciary relationship built through preoperative evaluation, consent, and operative care; and the intensivist’s claim, grounded in how intensive care is organized—and outcome data settle neither [187–193]. Survival after major surgery often hinges on prompt recognition and surgical treatment of postoperative complications, a compelling rationale to keep the surgeon embedded in postoperative care [194–196]. In framework terms, the underlying task conflict (continued rescue versus transition to comfort care) is usually legitimate and structurally generated, while process ambiguity about who decides produces a boundary dispute.

ICU organization varies by health system, and US-derived literature should be read accordingly. Closed, intensivist-led units are the international norm, with the US having the lowest proportion of closed units of any region [197]. Surgeon-reported conflict, when measured in the US, is highest in closed units that risk displacing the surgeon, whereas most surgical ICUs are collaborative or semi-open rather than fully closed [198, 199]. “Surgical buy-in” has not been tested as a named construct outside North America, though the underlying tensions appear elsewhere. A recent comparison between the US and France, where the family’s view is advisory, found the same core problems of inadequate advance directives, clinician moral distress over non-beneficial treatment, and value-based disputes about futility [200, 201].

### Conflict related to goals and transitions of care

The interface between intensive and palliative care can generate conflict over timing, framing, and communication style; palliative specialists use 55% fewer task-focused statements than intensivists and trend toward more relationship-building communication [22, 202]. The task conflict (when and how to introduce palliative care) is compounded by process conflict over whose communication approach governs family interactions, and by the identity threat that palliative involvement can pose to intensivists, who may read it as a judgment that their care, or primary palliative care, has fallen short [22, 202, 203].

Two structural choices can defuse this tension. First, distinguishing primary palliative care, the symptom management and goals-of-care conversations the ICU team provides itself, from specialty palliative care for complex or refractory cases reframes a consult as a routine adjunct rather than a verdict on the team, and it guards against the de-skilling that follows when specialists are treated as the only legitimate approach [204, 205]. Second, engaging specialists proactively through predefined screening criteria, rather than engaging them at a moment of disagreement, recasts their involvement as standard practice. Roughly one in seven ICU admissions meets such triggers, and in observational work trigger-based consultation has been associated with shorter ICU stays without affecting mortality [206, 207]. The apparent advantage favors targeted over blanket deployment: universal, specialist-led, prognosis-focused meetings for all high-risk families have not reduced family distress, and in one trial may have increased it [208].

Admission, triage, and discharge raise the same task-versus-process tension: disagreements about clinical appropriateness become entangled with disputes about authority [198, 209, 210]. Admission-decision research contrasts “critical illness exceptionalism,” which treats the ICU as a discrete domain with its own logic, with a “continuum of disease” view in which the ICU is one point on a longer trajectory, and the two perspectives can themselves be a source of conflict [211]. Consensus guidelines urge institutional policies that name decisional responsibility and a conflict-resolution pathway, yet more than a third of hospitals lack them [212, 213].

### Conflict across seniority strata

Hierarchy between senior physicians (attendings, consultants) and junior ones adds another potential source of conflict. Trainees, residents, and junior clinicians are often most continuously present at the bedside yet hold limited decisional authority. Qualitative studies link this directly to moral distress and professional detachment [214, 215]. An authority gradient can suppress trainee voice, with fear of conflict and retribution as notable barriers, worsened by institutional context [216, 217]. In the ICU specifically, Tong et al.’s secondary qualitative analysis found hierarchy shaping conflict around end-of-life care: subordinate clinicians became silent after being reprimanded, and senior physicians sometimes prohibited palliative care consultation [22].

This silence is better understood as context-dependent and threat-specific than as a default. Trainees speak up readily about familiar safety threats but far less about unprofessional behavior, with 20% willing to raise unprofessional behavior with an attending versus 71% for safety threats, and they weigh whether speaking up is both safe and likely to work [216, 218]. Perceived safety tracks less with profession than with how senior clinicians behave. On ICU rounds, attending inclusiveness is the dominant predictor of psychological safety, with no significant difference across clinician types, and the ability to challenge a superior is seemingly trainable rather than innate [219, 220].

The voice gradient is not only top-down; senior clinicians may also withhold candid judgments for fear of consequences. The “failure to fail” literature documents supervisors’ reluctance to record honest negative evaluations of underperforming trainees, citing anticipated appeals and repercussions, and faculty are similarly hesitant to report professionalism lapses [221–223]. A related concern, that learners’ evaluations and reports may deter senior clinicians from honest feedback, is increasingly voiced but thinly evidenced and may partly reflect misperception [224]. Whether such bidirectional constraints meaningfully shape ICU conflict is an open, under-studied question and a research priority.

### Causes and consequences

Three structural mechanisms help explain why team conflict persists. First, hierarchy and constrained voice can produce an absence of reported conflict that reflects suppression rather than harmony [225]. Second, exclusion from decisions amplifies perceived inappropriateness: when symptom-control decisions were made by physicians alone rather than shared with nurses, the odds of clinicians perceiving care as inappropriate were 73% higher [160]. Third, moral distress accumulates; Epstein and Hamric described a “crescendo effect” in which unresolved ethical concerns leave moral residue that lowers the threshold for the next episode [23]. The leading drivers of moral distress appear consistent across health systems; among Turkish ICU nurses, for example, the top source was poor intra-team communication [226]. An Indian expert consensus likewise names resolving conflict within the treating team, not only with families, as an explicit goal of end-of-life communication, tying both to moral distress and burnout [77].

The DISPROPRICUS study assessed ethical climate across 68 ICUs using a validated climate questionnaire [227, 228]. ICUs in the “good” climate cluster identified excessive care better and reached treatment-limitation decisions faster than those in the “poor” cluster, tying perceived inappropriateness to a potentially actionable organizational variable. The moral-distress concept has since been extended, and a dedicated consultation service has shown potential to modestly reduce situational moral distress, particularly among ICU staff [229, 230].

Conflict with colleagues is independently associated with higher burnout among intensivists, and among critical-care clinicians more broadly, high moral distress carries roughly two- to threefold higher odds of severe burnout [231, 232]. Burnout and moral distress are in turn associated with attrition and intention to leave: intensive care has among the highest intention-to-leave rates among US academic physicians, with relational and organizational factors predicting departure independently of burnout, and per-nurse turnover estimated at US $75,000 [159, 233–235]. At the patient level, a systematic review found poor communication contributed to 24% of patient safety incidents and was the sole cause in 10% [236].

## Matching interventions to conflict type and stage

Choosing an intervention without first identifying the conflict type and stage (whether conflict is emerging, established, or entrenched) risks effort aimed at the wrong cause, since an approach suited to early disagreement can fail once conflict has been established (Table 5) [237]. The evidence favors preventive, design-based interventions first, then early recognition and bedside de-escalation, with formal supports reserved for entrenched conflict (Table 6).

**Table 5:** Type-specific conflict diagnosis in an intensive care context

Conflict type	Features	Common ICU examples	High-yield first steps	When to escalate^*^
Task	Disagreement centers on facts, data interpretation, prognosis, or treatment options; parties can articulate opposing clinical positions	Resuscitation preference discussions, extracorporeal support candidacy, antibiotic duration, transfusion thresholds, discharge readiness assessment	Clarify the specific decision at stake. Make uncertainty explicit rather than masking it [250, 301]. Distinguish prognostic questions (factual) from values questions (preferential). Generate options; use time-limited trials to convert binary disputes into sequential decisions [263, 264, 302]	When task disagreement is stable and unresolvable through information alone, suggesting genuine value conflict requiring formal support
Process	“Who decided this?” or “nobody told us.” Confusion about authority, sequence, or communication pathway	Family excluded from rounds, consulting service overriding primary team, triage disputes over bed allocation, unclear ownership of postoperative patients	Clarify roles and decision authority. Explain the decision pathway transparently [46]. Structure family conferences, align before, and follow-up [152]. Ensure structured input from all professional groups [47, 48]	When process repair alone is not resolving the substantive dispute, or when process failures reflect structural problems requiring institutional change [53, 266]
Relationship	Personal attacks, avoidance, or distrust language: “they don’t care.” Parties unable to engage substantively	Family distrust of the clinical team, inter-team antagonism, inter-service rivalry, family or surrogate anger rooted in prior harm or institutional mistrust that has attached to the current relationship	Acknowledge emotion directly (NURSE framework) [249]. Name mistrust without becoming defensive [250]. Slow the pace. Explore interests beneath positions [43, 248]. Consider whether the conflict reflects structural inequity in communication or escalation	When relationship repair is needed before substantive clinical work can resume; when skilled facilitation or mediation is indicated, with ethics consultation reserved for coexisting value conflict [153, 154, 251, 254, 257–259]

^*^Escalation thresholds reflect a stage-matched principle: interventions that resolve early task or process disagreement lose traction once conflict hardens into the relationship domain [237]

This is an author-developed synthesis linking predominant conflict type to appropriate initial interventions. Note that many conflicts involve elements of more than one type; the goal is to identify the primary driver and match the response accordingly. ICU, intensive care unit; NURSE, Name Understand Respect Support Explore (a communication framework for responding to emotion)

**Table 6:** Evidence summary for key conflict-related interventions

Intervention	Key evidence	Primary findings	Strength of evidence	Implementation considerations
Structured interdisciplinary rounds [238, 240, 303]	Before-after, single center (Pronovost 2003); systematic review, 33 studies (Heip 2022); meta-analysis, 30 studies, > 32,000 patients (MacKinnon 2024)	Goal understanding improved from < 10% to > 95% with reduced ICU LOS (Pronovost). Systematic review finds positive trends for patient-centeredness and collaboration, but evidence quality is low and heterogeneous (Heip). Checklist meta-analysis suggests lower mortality (RR ~ 0.80, very low certainty; MacKinnon)	Low to moderate	Requires explicit input protocols for all disciplines. Effects depend on institutional culture and leader behavior. Structured scripts can improve nurse participation and collaboration processes
Interprofessional education and collaboration interventions [246, 247, 304, 305]	Cochrane reviews (Reeves 2013 and 2017); scoping review of interprofessional simulation-based team training (Bochatay 2025); Recommended approaches to education and team-based care (IPEC 2023)	Strongly endorsed and aligned with task-process-relationship framework via shared mental models, role clarification, and intergroup contact. Heterogeneity precludes firm inferences on behavior or patient outcomes. Few ICU programs comprehensively measure outcomes or conflict-specific outcomes	Low	Requires certain conditions (equal status, cooperation, common goals); can reinforce professional stereotypes when poorly designed. Sustained behavior change depends on concurrent structural change
Structured family conferences [72, 269–271, 274, 275, 306]	RCT, multicenter (Lautrette 2007); stepped-wedge cluster RCT, 1,420 patients (White 2018); cluster RCT, 34 ICUs, 875 relatives (Kentish-Barnes 2022); RCT, 385 patients (Kentish-Barnes 2024); cluster RCT, 16 ICUs, 885 family members (Naef 2025); RCT, 444 surrogates (Butler 2025); meta-analysis, 10 trials, 3,824 surrogates (DeForge 2022)	End-of-life support reduces prolonged grief (15% vs 21%, p = 0.035; Kentish-Barnes 2022). Communication interventions consistently improve process measures but generally do not reduce long-term surrogate psychological symptoms. PARTNER improved communication quality and reduced ICU LOS without reducing HADS scores. Four Supports (97% fidelity) was null on all psychological outcomes. Meta-analysis shows small pooled effects of uncertain stability	Strong for process outcomes and grief reduction at end of life; moderate to weak for long-term psychological symptom reduction	End-of-life specific protocols (three-step model including post-death meeting) show the clearest benefit. Nurse-led pathways can reduce costs and improve communication quality. Pre-conference team alignment is a critical but understudied component
Proactive ethics rounds (PEACE and similar) [243, 266, 307, 308]	Before-after, single center (Wocial 2017); before-after, 3 centers, 6 ICUs, 1,649 patients (Pavlish 2020); case series, 314 discussions over 9 years (Meyer-Zehnder 2021); SCCM clinical practice guideline (Marshall 2025)	Ethics rounds reduced LOS in a single-center study while improving moral distress among nurses only (Wocial). In multicenter replication: increased supportive-service consultations but LOS reduction not replicated (Pavlish). Weekly ethics discussions sustainable over 9 years (Meyer-Zehnder). SCCM guidelines recommend proactive consultation for patients who may no longer benefit from intensive care (low certainty)	Moderate (multicenter replication; guideline endorsement)	Requires dedicated time and facilitator. Embeds conflict prevention into routine workflow. Blanket proactive referrals appear less effective than targeted approaches. Benefits nursing staff disproportionately. Adaptable to specific high-acuity technologies (e.g., ECMO)
Ethics consultation [251–253, 259, 309, 310]	Multicenter RCT, 7 hospitals, 551 patients with value-related conflicts (Schneiderman 2003); Meta-analysis, 16 studies (Au 2018); Cochrane systematic review, 4 eligible studies (Schildmann 2019); scoping review (Bell 2022); retrospective/prospective cohort, 1,150 patients (Lin 2025); survey of top 50 US academic hospitals (Neal 2020)	Schneiderman RCT: among non-survivors, ethics consultation reduced ICU and ventilator days. Au meta-analysis: increased treatment consensus (fourfold), reduced ICU LOS (4.65 days), reduced non-beneficial treatment. However, Cochrane review assessed certainty as very low across all outcomes. Mandatory CEC policies associated with decreased ethical conflict incidence (Lin). Only ~ 50% of top US academic centers have mandatory policies (Neal). Outcome measures remain unstandardized (Bell)	Low to moderate (Cochrane: low certainty; supportive observational data)	Triggered consultation more effective than blanket referral. Institutional variation in availability and quality of ethics services is large. Mandatory policies can be sustained but adoption remains uneven. Standardized outcome measures are needed to advance the evidence
Early palliative care integration [155, 260, 262, 266, 311, 312]	Cluster randomized crossover, 199 patients (Ma 2019); RCT, 91 patients (Helgeson 2023); systematic review, 58 studies (Metaxa 2021); ESICM guideline (Kesecioglu 2024); SCCM guideline (Marshall 2025); implementation review, 84 studies (Meddick-Dyson 2024)	Early triggered consultation increased DNR/DNI transitions (50.5% vs 23.4%), hospice transfers, and reduced ventilator days and tracheostomies without mortality difference (Ma). Second RCT: higher family satisfaction (Helgeson). ESICM and SCCM guidelines recommend early integration for high-risk patients (low to moderate certainty). Screening can increase consultation volume without changing downstream outcomes	Strong (oncology); moderate to strong (general ICU)	Most effective when trigger-based and integrated early rather than crisis-driven. Timing and framing remain sources of inter-service conflict. Substantial gap between effectiveness evidence and implementation knowledge. Evidence predominantly North American
Bioethics mediation [252, 265, 313]	Pre–post with 12-month follow-up (Zweibel 2008); systematic review of medical mediation (Lioupi 2025); complex intervention framework (Schildmann 2019)	Durable skill transfer at 12 months after conflict-resolution skills training for physicians (Zweibel). No new controlled studies since 2008. Systematic review supports mediation for healthcare dispute de-escalation but is not ICU-specific	Low (persistent absence of controlled outcome studies)	Requires trained mediators. Skills can be taught to existing ethics consultants. This remains the weakest evidence category among established interventions
Time-limited trials [263, 264, 266, 302]	Multi-hospital QI before–after, 209 patients (Chang 2021); SCCM guideline endorsement (Marshall 2025); narrative review synthesizing 18 empirical studies and 15 ethical analyses (Kruser et al. 2024); consensus definition with 16 essential elements via modified Delphi (Kruser et al. 2024)	TLTs as default care-planning led to earlier family meetings, reduced ICU/hospital LOS, and reduced invasive treatments without mortality change; greater reductions among decedents (Chang). Used in 8% to 27% of ICU patients. 65% of intensivists report familiarity, but implementation is variable and often incomplete; key elements such as end-of-trial decision discussions are frequently omitted	Low to moderate (single pre–post QI study without RCT; remainder is observational, qualitative, survey-based, and modeling work; no completed RCT to date)	Operationally distinct from generic family conferences and fair-process pathways. Frames treatment as time-bounded with pre-specified reassessment criteria, a conflict mitigation mechanism. Standardized framework published with evidence predominantly from US academic ICUs
Fair-process pathway for potentially inappropriate treatment [53, 266, 314, 315]	Multisociety consensus (Bosslet 2015); SCCM guideline (Marshall 2025); prospective observational, 533 patients (Hwe 2020); focus groups, 39 community participants (Neville 2020)	Seven-step structured escalation for potentially inappropriate treatment. SCCM guidelines recommend institutional policies to address such disputes (low certainty). Clinician recognition of non-beneficial treatment does not reduce its administration without structured processes (Hwe). 69% of community members had never heard of “futile care” (Neville)	Consensus-based; low certainty (SCCM guideline)	Provides procedural justice framework. Implementation depends on institutional infrastructure and legal context. Clinician-public communication gaps require attention. Equity implications of escalation tools warrant oversight
Pre-conference team alignment [21, 269, 270]	Expert recommendation; consistent with/incorporated within interprofessional communication pathways studied in PARTNER and FICUS (Bruce 2015, White 2018, Naef 2025)	Reduces mixed messaging and prevents intrateam disagreement from reaching families. No standalone empirical evaluation exists. Operationalized as a core component within multi-component interventions, but isolated effect remains unstudied	Low (expert recommendation; indirect evidence only)	Low cost and highly actionable. Addresses the mechanism by which team conflict converts to family conflict. Persistent absence of a standalone implementation trial is a notable evidence gap
Structured psychological support for surrogates [279, 306]	Pilot RCT, 60 surrogates (Lichtenthal 2024); meta-analysis of surrogate interventions, 10 trials, 3,824 surrogates (DeForge 2022)	EMPOWER, a pilot RCT of a brief (~ 2-h) cognitive-behavioral intervention that generated preliminary signal of effect on grief, PTSD, and depression at 3 months; confirmation awaits larger trials. Meta-analysis of communication-based surrogate interventions found only small, unstable pooled effects on psychological outcomes	Low (pilot RCT); high conceptual priority	Targets psychological mechanisms (experiential avoidance, coping, emotional processing) rather than communication processes. Deliverable within the ICU stay but needs further validation. Communication improvement and psychological support appear complementary, not redundant
Ethical climate cultivation [31, 228, 242, 316, 317]	Multicenter survey, 68 ICUs, 2,992 clinicians (Van den Bulcke 2018; Jensen 2019); stepped-wedge cluster RCT (Benoit 2024); cross-sectional, US setting (Silverman 2022); COVID-era study, 84 Dutch ICUs (Donkers 2021)	Ethical decision-making climate varies substantially across ICUs and predicts intent to leave. Physicians rate climate more favorably than nurses. The CODE trial, as the first ethical climate RCT, demonstrated that structured coaching improved goal-oriented care decisions, although it did not improve ethical climate	Moderate (first interventional RCT; strong associational data)	Modifiable through leadership behavior, institutional practices. Structured coaching is a feasible model. Complements psychological safety cultivation

CEC, clinical ethics consultation; DNI, do not intubate; DNR, do not resuscitate; ECMO, extracorporeal membrane oxygenation; ESICM, European Society of Intensive Care Medicine; HADS, Hospital Anxiety and Depression Scale; ICU, intensive care unit; LOS, length of stay; PTSD, post-traumatic stress disorder; QI, quality improvement; RCT, randomized controlled trial; RR, relative risk; SCCM, Society of Critical Care Medicine; TLT, time-limited trial

### Design and prevention

Design interventions keep disagreement focused on the task. Structured interdisciplinary rounds with explicit multidisciplinary input aim to build goal concordance, though the evidence is heterogeneous, with a large multicenter trial showing no benefit and a meta-analysis rating certainty as very low (Table 6) [238–240]. Two low-cost levers target specific transitions. Role clarity—specifying who decides what—keeps process conflict from turning relational, and pre-conference team alignment, surfacing disagreement before meeting the family, prevents mixed messaging. Standardizing recurrent decisions does similar work: default pathways, predefined escalation and de-escalation triggers, and time-limited trials with pre-specified reassessment settle routine choices in advance and supply objective criteria that anchor principled negotiation [43, 46]. The force of objective criteria depends on prior agreement: standards resolve an impasse only when the parties accept them as legitimate, and a rule imposed without that agreement tends to relocate the dispute onto the rule itself [241].

Cultivating ethical climate may add further benefit. Climates marked by mutual respect and open interdisciplinary reflection are associated with lower intent to leave, which may raise the threshold for escalation, though the CODE trial found climate hard to shift through physician coaching alone, pointing to team-level rather than individual interventions [234, 242]. Targeted approaches appear to outperform blanket ones: weekly ethics-facilitated goals-of-care rounds for long-stay patients reduced severity-indexed length of stay in the pediatric ICU (Table 6), whereas a non-targeted proactive ethics intervention showed no benefit [243, 244]. The HELLO trial, across 370 ICUs in 60 countries, found that a brief positive-communication intervention reduced burnout and improved ethical climate and intent to leave but did not reduce reported interprofessional conflict, which suggests that climate-focused and structural interventions act at different points on the same causal pathway [245]. Interprofessional education is widely endorsed and mechanistically aligned, but heterogeneity precludes firm inferences about outcomes (Table 6) [246, 247].

### Recognition and proximate de-escalation

At early stages, prompt recognition and bedside de-escalation matter. The first step is to identify whether the disagreement is about what should be done (task), how decisions are made (process), or damaged trust (relationship). For task and process conflict, returning to interests rather than positions is especially useful [248]. Eliciting interests depends on often-neglected prerequisites, chief among them emotional acknowledgment. Empathic communication frameworks such as NURSE (Name, Understand, Respect, Support, Explore) help uncover values and pace the conversation [249]. Asking early about prior negative healthcare experiences can similarly surface inherited mistrust before it is misread as conflict arising within the current relationship [25]. Naming prognostic uncertainty can reduce escalation by signaling transparency, though uncertainty disclosed without a plan for revisiting it can undermine confidence [250].

### Formal support

When conflict is entrenched, formal support should be matched to the dispute. Ethics consultation is associated with improved consensus, though its effect on length of stay is inconsistent and the evidence is of very low certainty [251–253]. It is best suited to genuinely ethical questions, such as conflicts of values, rights, and obligations, and the field frames the consultant’s role as “ethics facilitation,” helping parties clarify values and identify a justifiable range of options rather than imposing a decision [254]. In practice, though, consultations are often triggered by conflict and emotion rather than moral puzzlement: more than half arise from conflict-laden situations, and about three-quarters involve staff or staff-family disagreement [255, 256]. Ethics consultation is thus frequently asked to do conflict-resolution work it was not designed for. Where a dispute is fundamentally relational, mediation offers a distinct, interest-based pathway, and a growing argument holds that purely interpersonal conflict belongs with mediation or patient-relations services rather than clinical ethics [153, 154, 257, 258]. None of this makes consultation ineffective where it fits: in a multicenter randomized trial among ICU patients with value-related conflicts who ultimately died, it reduced nonbeneficial ICU and ventilator days without affecting mortality [259]. The lesson is to triage support to the problem: ethics consultation for value conflicts, mediation or facilitation for relational impasses.

Other supports act at specific points. Early palliative care started by predefined triggers reliably increases comfort-focused transitions in randomized trials, though length-of-stay effects are mixed [260–262]. Time-limited trials frame intensive treatment as time-bounded with pre-specified reassessment, with preliminary evidence of reduced nonbeneficial treatment [263, 264]. Conflict-resolution training participants reported durable skill transfer at 12 months, though without later evaluation [265]. For potentially inappropriate treatment, a fair-process pathway lends procedural legitimacy even when the outcome stays disputed, as now endorsed by relevant guidelines [53, 266].

### Proximal versus distal outcomes

A clinically important pattern runs across these support systems: they reliably improve proximal outcomes (comprehension, satisfaction, communication quality) but generally do not improve distal bereavement outcomes such as long-term anxiety, depression, post-traumatic stress, and prolonged grief, and several have shown no benefit or harm. Trials of information leaflets, question prompts, nurse-led support, and facilitated family conferences fit this pattern, including one delivered with 97% fidelity (Table 6) [267–272]. One randomized intervention intended to support bereaved families had the opposite effect: a clinician condolence letter increased depression and post-traumatic stress symptoms at six months [273]. The exception is support aimed at the dying process itself: a proactive end-of-life conference with a bereavement brochure, a three-step strategy for relatives’ presence around the death, the option of family presence during resuscitation, and ICU diaries have each reduced post-traumatic stress or grief [274–278]. Communication and conflict interventions should therefore be justified by their proximal benefits, not offered as prophylaxis against grief, which is shaped largely by the death itself. Early evidence suggests that interventions supporting surrogates’ own coping may begin to address the distal gap [279].

## Implementation and future directions

Violence can be seen as the terminal failure mode of conflict management, though the link must be drawn carefully. ICU workplace violence is common, with about 31% of staff reporting physical and 57% verbal violence, but it is predominantly patient-perpetrated and driven by delirium and agitation rather than interpersonal conflict; in a point-prevalence study confusion was the leading contributor, and visitor or family episodes were not captured [280, 281]. Conflict-driven family aggression is a distinct pathway, and the evidence is thin: the claim that unresolved conflict escalates to violence rests on juxtaposing separate prevalence literatures rather than any study testing the link, so it is best treated as a hypothesis and research priority [282, 283].

The available responses are weakly evidenced and not neutral. Flexible visitation reduces delirium and family anxiety without raising mortality or infection, so visitor restriction as a disciplinary tool is hard to justify, and coercive responses carry equity risk: emergency department physical restraint and security responses fall disproportionately on Black patients, a pattern under-studied in the ICU and unexamined outside the US [284–286]. Escalation tools such as behavioral contracts, security involvement, and visitor restrictions therefore warrant the same institutional oversight as other high-stakes decisions, and used without clear criteria they risk introducing bias [287–292]. The distinction between conflict and safety threat must be maintained: ordinary disagreement about goals should not be securitized, which delegitimizes families advocating for their values, whereas genuine safety threats need their own protocols and de-escalation training.

Implementation should fit local context. Units with high burnout may gain most from ethical-climate measurement and moral-distress support; units where complaints center on inconsistent messaging should prioritize pre-conference alignment; units with inter-service friction need explicit role clarity at boundaries. Strategy should also track the local legal architecture for decisions on behalf of incapacitated patients: interventions built around default-surrogate hierarchies may need adaptation in best-interests or benefits-based jurisdictions, where formal responsibility, and thus the target for process intervention, rests with the clinical team. Where marginalized populations are served, embedding resourced cultural liaisons in the ICU team is a structural investment of the same kind; clinicians describe such roles as fundamental to bridging communication while also cautioning that under-resourced or lone appointments can invite tokenism and burnout [76]. Sustained improvement requires discrete, well-scoped interventions and protected leadership time and resources, not just skills training [293]. Economic analysis is sparse, but per-nurse turnover costs and the climate-retention link suggest conflict-management infrastructure may pay for itself through retention alone [159, 233–235].

Synthesized evidence is also subject to drift as case mix, care structures, measurement, and societal norms shift beneath published findings, so before transporting any recommendation clinicians should ask whether the patients, structures, and norms match those under which the evidence was produced [294–296]. The field would benefit from validated conflict-specific measures, trials targeting conflict resolution as a primary rather than secondary outcome, study of trainee-mediated conflict and the hidden curriculum, and equity-disaggregated reporting across interventions, including whether the stigmatizing-documentation, coercive-escalation, and mistrust dynamics documented largely in US settings generalize across the hierarchies salient elsewhere. Evaluation should ask not only whether an intervention reduces aggregate conflict but whether participation, perceived respect, and coercive responses differ across locally relevant groups, since aggregate improvement can conceal unchanged or widened inequities. The literature on groupthink and premature consensus is strikingly thin despite hierarchy and pressure for team harmony being key drivers inherent to intensive care, which may reflect methodological difficulty or a reluctance to study dynamics on which current team structures depend. Post-conflict restoration is likewise nearly absent from ICU quality literature: team debriefing after morally difficult conflicts, explicit processing of moral distress, and service recovery with families could each interrupt the crescendo effect, within a restorative culture oriented to learning rather than fault-finding [297].

## Conclusions

Family and team conflict in the ICU are not separate problems but intertwined ones, stemming from how units handle disagreement under uncertainty, high moral stakes, and distributed authority. The literatures on moral distress, psychological safety, ethical climate, and collaboration look distinct in isolation, yet they measure the same thing through different lenses: how institutions and individuals process competing values and irreducible uncertainty. Integrating them reveals both the pervasiveness and the modifiability of ICU conflict.

When conflict arises, the aim is not to suppress disagreement but to recognize its type and intervene before it migrates from the task and process domains into the relationship domain, where it becomes entrenched and destructive. Individual communication skill cannot reliably overcome structural failure. System design with attention to structured rounds, role clarity, pre-conference alignment, ethical-climate cultivation, and equitable escalation is the more durable investment. These measures restore the conditions on which trust depends: acknowledgment, respect, integrity, and accountability. Disagreement in intensive care is inevitable; destructive conflict is not.

## Data Availability

No datasets were generated or analysed during the current study.

## Abbreviations

APP: Advanced practice provider
ICU: Intensive care unit
US: United States
